# Bulb Cannula Safety for Breast Fat Grafting

**DOI:** 10.1093/asjof/ojaa014

**Published:** 2020-04-13

**Authors:** Marcos Sforza, Nicole Martinez, Nathalia Araujo, Roberto de Mezerville, Jose Andrés Castro

**Affiliations:** 1 Elective Internship in Plastic Surgery, Dolan Park Hospital, Birmingham, England, UK; 2 Industrial Designer and RDI Engineer at Establishment Labs, Alajuela, Costa Rica; 3 Chemical Engineer and RDI Manager at Establishment Labs, Alajuela, Costa Rica; 4 Industrial Engineer and Chief Technology Officer at Establishment Labs, Alajuela, Costa Rica; 5 Medical Affairs Coordinator at Establishment Labs, Alajuela, Costa Rica

## Abstract

Autologous fat transfer is a common technique to refine the contour of the breast after prosthetic augmentation or reconstruction, correcting remaining asymmetries by injecting previously harvested fat tissue with a cannula. Current procedures are often performed without visualization of the cannula at the delivery site and may require subsequent verification of the implant’s integrity. The present paper aims to evaluate the safety of a new bulb tip cannula to be used during breast implant procedures for injecting fat adjacent to a breast implant that reduce the risk of damaging the implant. Two conventional cannulae and 3 bulb cannulae, which have an atraumatic distal tip, were tested in a simulated implant-puncture setting in 3 positions (at 0°, 45°, and 90° of incidence). A Tensile Tester (Instron, High Wycombe, UK) was used to apply force with each cannula device and record the amount of force applied in the attempt to penetrate the implants used, with shell layers having a variable thickness. No implant rupture was observed with the bulb tip cannulae regardless of size or the position in which the cannulae were pressed against the implants. The cannula opening was not impeded and tended to bend instead; 27% of the cases with the conventional lipo-cannulae caused an implant rupture. The bulb tip cannula could enhance the safety of the fat transfer procedure by ensuring no iatrogenic implant disruption and optimal delivery of the fat tissue.

Many patients receive implants for medical and/or aesthetic purposes. For example, breast augmentation with implants is a common procedure in many parts of the world and is the top aesthetic surgery performed in the United States.^1^

Often, women who have suffered from breast cancer or mammary hypoplasia (eg, due to a lack of, or damage to, mammary tissue) opt for silicone gel-filled breast implants as part of their reconstruction. After such surgery, it is typically necessary to refine or supplement breast shape even further to correct any remaining deformities or asymmetries. In such cases, superficial reconstructive methods, such as fat grafting, can be effective.

During fat grafting, the surrounding breast area is enlarged or filled by injecting autologous fat through an incision with a cannula. The cannula opens a channel as it is inserted through the incision, and when it is pulled back, the released fat fills up the opened spaces. However, care must be taken to avoid touching the outer surface of the breast implant with a sharp distal end of the cannula, as doing so could endanger the implant’s integrity. Puncturing or scarring the implant shell could cause it to rupture, which in turn could provoke health and aesthetic risks.

Iatrogenic injury of an implant with surgical instruments can result in its rupture.^2^ Thus, without careful handling, sharp tools regularly used during implantation can cause device failure. Although reports of breast implant puncture due to lipofilling cannulae are infrequent,^3^ cannula use remains a risk to implant integrity.

Current procedures are often performed without visualization of the cannula at the delivery site, and thus, after typical fat injection procedures, it is difficult to verify that the implant was not damaged during injection, particularly in cases of silent implant rupture.

This paper aims to evaluate the safety of a new bulb tip cannula for injecting fat adjacent to a breast implant—primarily, its reduced risk of damaging the implant. This newly developed special cannula (with pending US patent application number 16/136,400) has a lumen therein, an atraumatic distal tip, and an opening disposed proximal to the distal tip.

## METHODS

Studies were conducted at Establishment Labs (Alajuela, Costa Rica) between January and March 2016 to evaluate the performance characteristics of the new bulb tip cannulae in comparison to existing fat grafting cannulae. The method of testing involved inserting a conventional cannula and the bulb tip cannula into different sizes of breast implants (*n* = 6) to simulate implant puncture that could occur while performing fat grafting adjacent to its surface. The bulb tip cannula is configured to apply at least 25 N of compressive force, such as from 25 N to about 40 N of compressive force, to a breast implant shell without puncturing it. The force used to rupture the implant was measured. For these tests, an Instron Tensile Tester (Instron, High Wycombe, UK) was used to apply force with each cannula device and record the amount of force applied to different breast implants. The Tensile Tester uses pneumatic, mechanical, and electronic systems to transmit measurement data to a computer. The cannulae used in these studies were placed in the upper grip of the Tensile Tester. The equipment used for the studies was configured to provoke the cannula to penetrate the implant and measure the force applied. The distance that the cannulae pressed into the implant was found to be dependent upon implant type and its orientation in the Tensile Tester relative to the cannula (Video 1). 

### Bulb Cannulae Study

**Figure 1.**
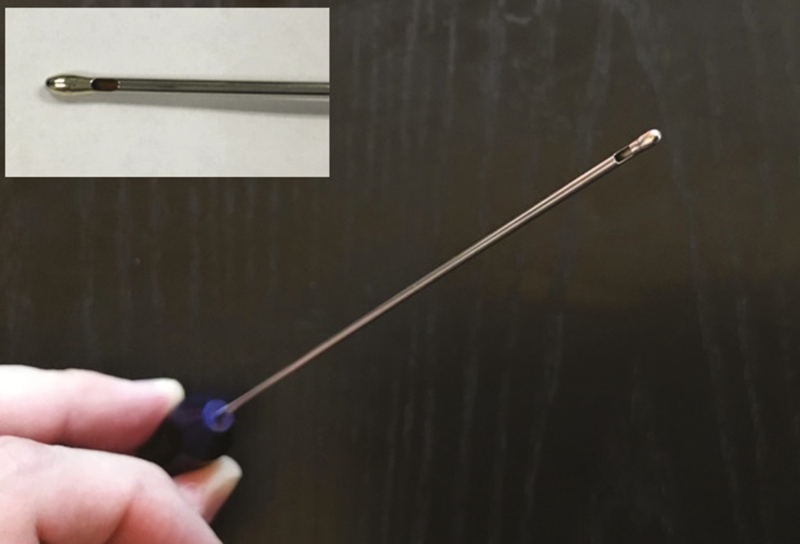
Example of the bulb cannula in detail.

A first compression test was performed using bulb cannulae (cannulae 1-3) to apply force against 6 silicone gel-filled implants (implants 1-6). Cannulae 1 to 3 were each stainless steel having an ovoid, bulbous-shaped tip. Cannulae 1 and 2 each had a length of 120.0 mm, an outer diameter of 1.60 mm, and a rounded rectangular opening with a length of 2.50 mm and a width of 1.20 mm. The distance from the distalmost tip of cannulae 1 and 2 to the edges of their respective openings was 5.5 mm. Cannula 3 had a length of 150.0 mm, an outer diameter of 2.10 mm, with a rounded rectangular opening having a length dimension of 3.20 mm, and a width dimension of 1.60 mm. The distance from the distalmost tip of cannula 3 to the opening was 7.9 mm (Figure 1).

The implants tested were silicone gel-filled Motiva Implants (Establishment Labs, Alajuela, Costa Rica). Implants 1 to 3 were Motiva Ergonomix implants with SmoothSilk/SilkSurface (Establishment Labs, Alajuela, CR) nanosurfaces. Implants 4 to 6 were Motiva Round implants with SmoothSilk/SilkSurface (Establishment Labs) nanosurfaces.

The silicone gel used in implants 1 to 3 (ProgressiveGel Ultima, Establishment Labs) had an elasticity that allowed the projection of the implants to shift in response to gravity, simulating the ergonomics/movement of natural breast tissue. As indicated by the lower penetration value, the silicone gel used in implants 4 to 6 (ProgressiveGel Plus, Establishment Labs) had somewhat greater cohesion, such that the gel was less responsive to gravity. The implant volumes were 925 cc for implants 1 and 4; 375 cc for implants 2 and 5; and 105 cc for implants 3 and 6. The safety and benefits of Motiva Implants have been discussed in several other papers.^4-6^

**Figure 2.**
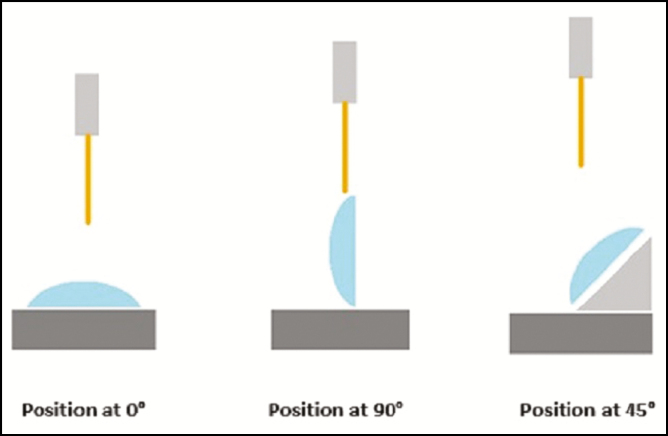
Implant positions during the test.

**Figure 3.**
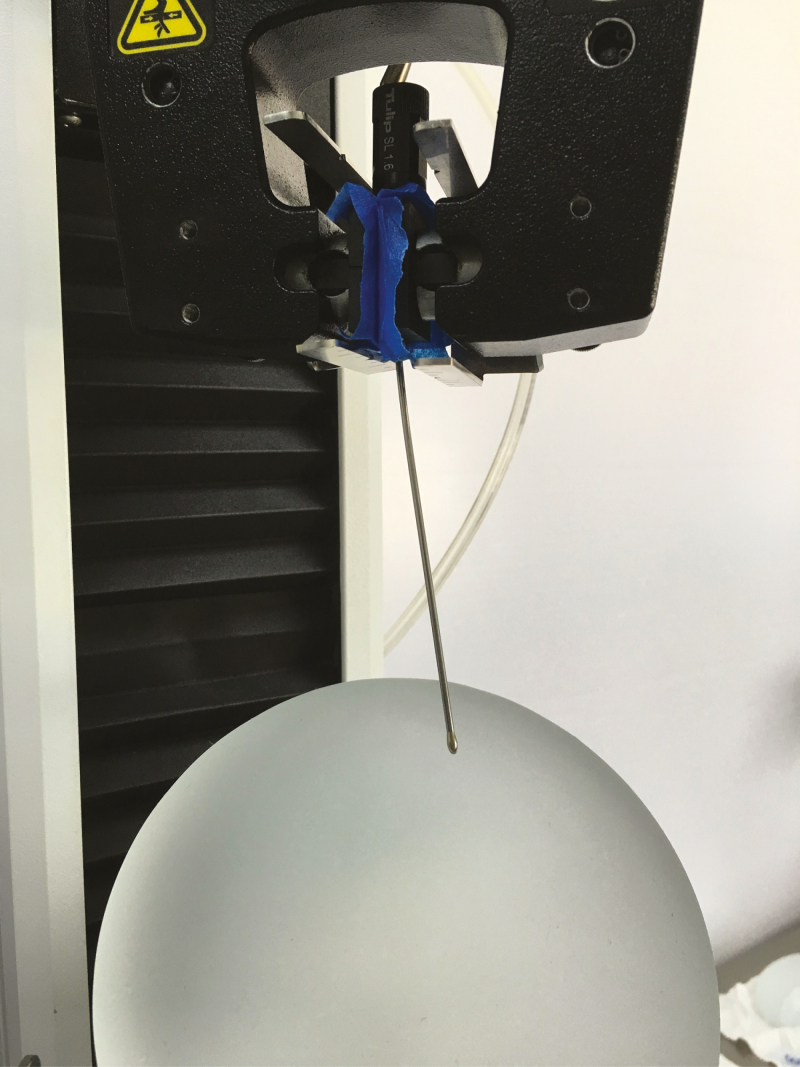
Implant positioned at 45° during the test with the bulb tip cannula.

The compression test was executed under various parameters (eg, implant rheology, implant volume, and orientation within the Tensile Tester at 0°, 45°, and 90°, as shown in Figures 2 and 3) to identify under which conditions implant rupture may occur upon contact with each cannula. The study was performed by placing each implant in horizontal, vertical, and angled orientations on a flat surface. For each orientation, the cannula was placed in the upper grip of the Tensile Tester and moved toward the implant to touch its surface. The amounts of force applied to the implant through the cannula were gradually increased.

In a horizontal position at 0°, the cannula was moved along the projection of the implant, perpendicular to its base. At 45°, the implant was supported on a plate with a 45° angle, and the cannula was moved from the apex of implant toward its base. In a vertical position at 90°, the cannula was moved along the total base diameter of implant.

Testing was carried out as follows. To start, each cannula was placed in the upper grip of the Tensile Tester with the distal end of the cannula almost touching the surface of implant. Before initiating the test, the distance from the cannula’s distal end to the base below the implant was measured by removing the implant and manually moving the upper grip with the cannula to the base and recording the distance. After this distance was obtained, the cannula was moved back to its initial position, and the Tensile Tester was configured to travel the measured distance. During the test, the Tensile Tester moved the upper grip downward, enabling the cannula to push down on the surface of the implant. As this downward movement occurred, the Tensile Tester recorded the force applied against upper grip (ie, the resistance of the implant).

### Conventional Cannulae

**Figure 4.**
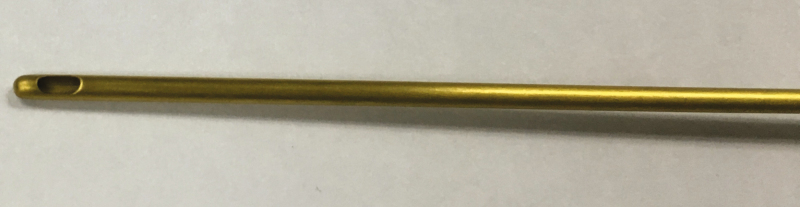
Example of a conventional cannula in detail.

In a separate compression test, 2 comparison fat grafting devices (cannulae 4 and 5) without bulbous tips were tested against implants 1 to 6 under similar conditions. These comparison fat grafting devices were formed of stainless steel (Figure 4). Cannula 4 had a length of 120.0 mm, an outer diameter of 1.65 mm, an inner diameter of 1.36 mm, an opening with a length A of 2.6 mm and a width B of 1.1 mm, and a length of 4.2 mm from the distal end of its opening to its distalmost tip. Cannula 5 had a length of 150.0 mm, an outer diameter of 2.11 mm, an inner diameter of 1.7 mm, an opening with a length A of 3.3 mm and a width B of 1.3 mm, and a length of 2.7 mm from the distal end of its opening to its distalmost tip.

## RESULTS

**Figure 5.**
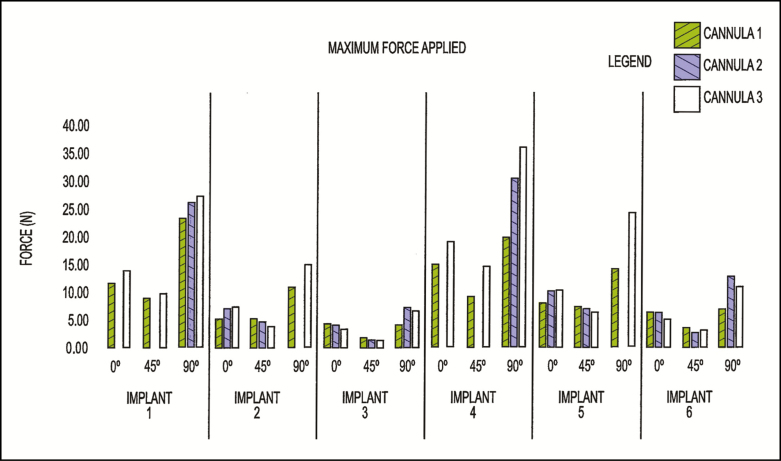
Summary of the test findings of the bulb cannula.

Results for cannulae 1 to 3 with atraumatic distal tips are shown in Figure 5. Maximum resistance of each implant to the force applied by the cannula was measured as maximum force by the Tensile Tester sensors. This force tended to increase for implants with higher volumes (eg, implants 1 and 4) or when the traveling distance of the cannula increased. For example, at 90°, more gel lay between the cannula and the base than in any other orientation. The force applied by cannula 3 was generally higher than the force applied by cannulae 1 and 2. This was understood to result from the larger outer diameter of cannula 3 (2.10 mm) as compared with the outer diameters of cannulae 1 and 2 (1.60 mm). Thus, the bulbous shape tip of cannula 3 was also larger, resulting in more surface contact and resistance between cannula 3 and the implants. It was also found that implants 1 to 3 with their less cohesive silicone gel (such that the gel was more responsive to gravity to simulate the movement of natural tissue) had higher values of maximum force applied by the cannulae, which may be due to the ability of the gel to deform around the cannula as it pressed against the implant surface.

No ruptures were observed with the bulb tip cannulae. The various distal tips of cannulae 1 to 3, regardless of size or the position in which the cannulae were pressed against the implants, did not penetrate the outer shells of the implants. Instead, the distal tip pushed gently against the surface of the implant, and the implant’s resultant deformation did not impede or block the opening in the cannula. The results of the bulb tip cannulae are shown in Figure 5.

**Figure 6.**
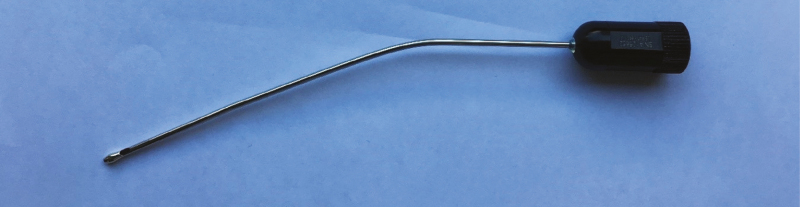
Bended bulb cannula after compression test with RSC-925 at 90°.

Cannulae 1 and 2 were identical. Because of their relatively thin width, cannulae 1 and 2 tended to bend when compressed against the implants of higher volume (375 and 925 cc). That is, rather than release the pressure against the implants by puncturing their shells, the cannulae bent. In 1 case, cannula 2 bent so severely that it became unsuitable for further testing (Figure 6). Results for cannula 2 are, therefore, not shown for some orientations of implants 1, 2, 4, and 5. Cannulae 1 and 3 were used to collect data for each of implants 1 to 6 in all 3 orientations.

Further testing of cannulae 1 to 3 showed that 35 N or greater compression force can be sustained without damage to the implants. The distal tips of cannulae 1 to 3 were found to be capable of pressing into the implant surfaces at a distance approximately equal to the diameter of the implant’s base, or its projection height, without damaging the implants. It was found that 27% of the cases with the conventional lipo cannulae 4 and 5 in this comparison test failed, causing ruptures among the implant shells.

**Figure 7.**
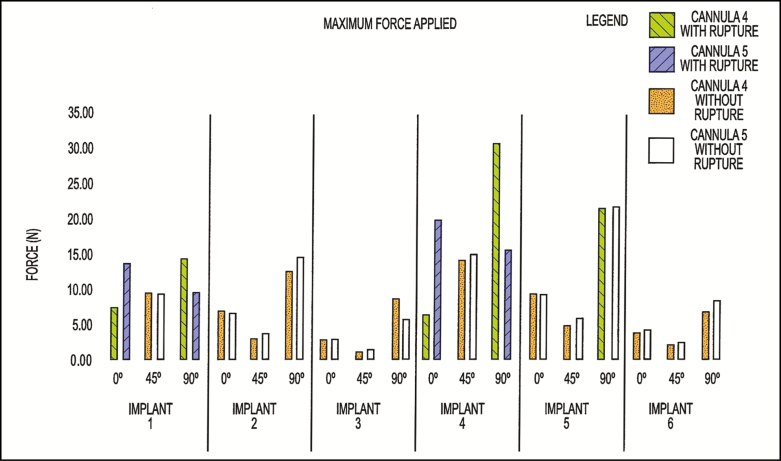
Summary of test findings with the conventional cannula.

Despite having rounded tips, each of the comparison fat grafting devices included an exposed injection hole relatively close to the end of the cannula. The sharp edges of these holes caused ruptures and/or tears of the implant shell when sufficient force was applied through the cannulae to press against the implant. A summary of the test findings of the conventional cannulae can be found in Figure 7.

In all tests, the force was not increased manually by the operator, as the process is preconfigured to measure the resistance of each implant and record the maximum force applied (maximum resistance). For the cannulae that did not rupture the implant, this maximum force was found to occur when the cannulae exceeded 95% of the penetration distance. For the cannulae that caused implant rupture (ie, the non-bulbous ones), maximum force occurred at the point before the cannula pierced or ruptured the implant.

## DISCUSSION

After more than 40 years since its inception, liposuction is currently one of the most accomplished aesthetic interventions around the world due to simple surgical techniques and very low complication rates. Moreover, lipofilling is a widely used technique in several different clinical situations, such as correction of asymmetry and defects in the body’s profile, loss of volume, or retrograde or atrophic scars; or in regenerative medicine for the treatment of chronic wounds.^7^ It is important to say that this study did not analyze if the bulb tip cannula has less penetration risk than a conventional one in regard to the implant scar capsule and further studies in this area are encouraged. In this new era of composite procedures involving silicone implants and fat grafts, the safety of fat grafting cannulae is paramount.

## CONCLUSION

As an adjuvant procedure in breast aesthetics and reconstruction, fat grafting is technically challenging, especially as it is performed without direct or tool-assisted visualization of the implant. The multiple sessions of fat grafting that typically occur per case also increase the overall risk due to repeated occurrences of these circumstances. Given these circumstances, effective and protective measures must be taken to avoid silent implant rupture. In this paper, the author has proven the safety of a new bulb tip cannula in its use for fat grafting alongside silicone breast implantation.

## Disclosures

M.S. is a paid consultant, shareholder, and member of the Medical Advisory board at Establishment Labs. N.M. was an employee of Establishment Labs at the time of the manuscript preparation. N.A. and J.A.C. are employees of Establishment Labs. R.M. is the Chief Technology Officer of Establishment Labs. M.S., N.M., N.A., and R.M. are authors of the patent but do not receive any royalties for this invention. Establishment Labs RDI conducted all the engineer tests presented on the paper.

## Funding

Establishment Labs funded the publication costs of this manuscript.
